# Celebrating 20 years of progress: accelerating towards elimination

**Published:** 2018-11-09

**Authors:** Virginia Sarah, Aparna Barua Adams, Tim Jesudason

**Affiliations:** 1 Global Partnerships Executive: Fred Hollows Foundation and Immediate Past Chair: International Coalition for Trachoma Control, London, UK.; 2 Project Manager: International Coalition for Trachoma Control, London, UK.; 3 Special Projects and Campaigns: International Coalition for Trachoma Control London, UK.

**Twenty years after the World Health Assembly adopted a resolution that targeted trachoma for elimination, work is continuing at a rapid pace.**

**Figure:**
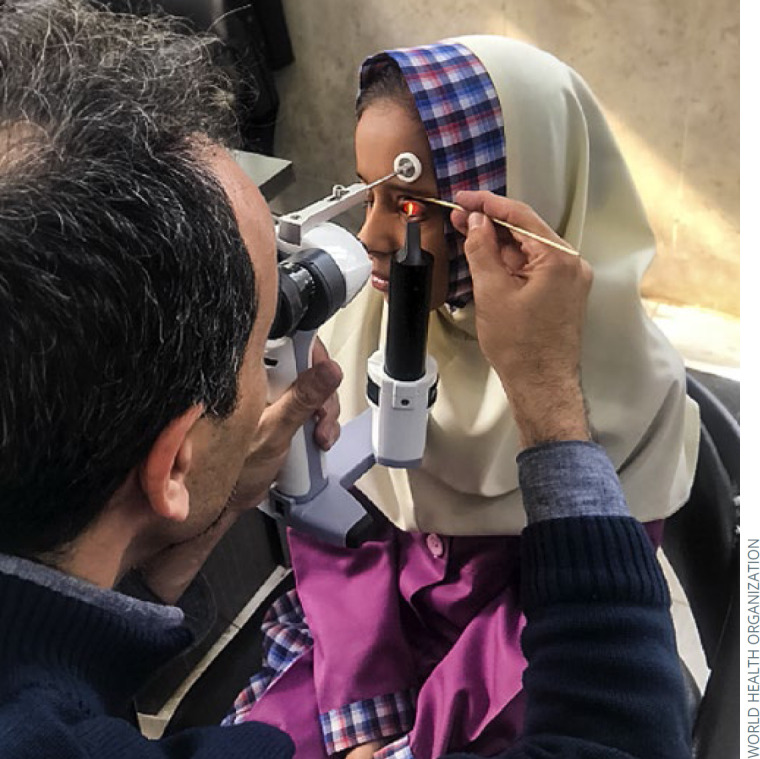
The Islamic Republic of Iran has just become the eighth country to be declared by the World Health Organization as having eliminated trachoma.

Trachoma is the world's leading infectious cause of blindness and one of twenty neglected tropical diseases (NTDs) that affect over one billion of the world's poorest people. In 1998, the World Health Assembly adopted Resolution 51:11,^1^ which targeted trachoma for global elimination. Since then, progress has accelerated.

The number of people at risk of trachoma has more than halved since 2011, thanks to the efforts of stakeholders in the WHO Alliance for the Global Elimination of Trachoma by 2020 (GET2020). Since 2012, eight countries have been validated by WHO as having eliminated trachoma as a public health problem: Oman in 2012, Morocco in 2016, Cambodia, Lao Peoples Democratic Republic and Mexico in 2017, and Ghana, Nepal and the Islamic Republic of Iran in 2018. By the year 2020, it is estimated that at least 70% of endemic districts will have reached the WHO target for elimination as a public health problem: prevalence of trachomatous inflammation-follicular (TF) 1–9 below 5%.

This year's data^2^,^3^ demonstrates the significant global and regional progress made during 2017 and early 2018.

- The number of people at risk of trachoma, because they live in districts where trachoma is endemic, has decreased from 182 million people in 2017 to 157.7 million as of April 2018
- Last year, 231,447 people received surgery for trachomatous trichiasis and 83.5 million were treated with antibiotics
- A total of 140 new districts in endemic countries received antibiotics for the first time in 2017. At the same time, it was reported that 250 districts reached their elimination targets, resulting in 27 million people no longer requiring antibiotic treatment
- For the first time, we are now providing antibiotics to over 50% of the people who need it.

Recently, stakeholders completed the Global Trachoma Mapping Project (2012–2016), which clearly identified the global prevalence of trachoma and helped us to define the actions and resources needed to eliminate trachoma.

The rapid progress shown in the global programme demonstrates that trachoma can be eliminated as a public health problem. However, to achieve global elimination, every endemic community must be reached with treatment interventions. Ethiopia has the greatest need, with 70 million people living in areas where trachoma is endemic (this is 44% of the global population currently at risk of trachoma).

To meet our goals, the most marginalised of people – indigenous and nomadic tribes, refugees, internally displaced people, and people living in conflict areas – must be reached. Sustained political will, operational research, new donors and partnerships, as well as financial and human resources will be essential to continue progress towards elimination.

In recent years, progress in the implementation of the WHO-endorsed SAFE strategy (Surgery for trichiasis, Antibiotics, Facial cleanliness and Environmental improvement) has been marked by unprecedented partnerships and coordination among donors, implementing organisations and ministries of health, guided by the GET2020 elimination roadmap, Eliminating Trachoma: Accelerating Towards 2020.^4^

**“There are 157.7 million people in 43 countries who are at risk of trachoma.”**

The SAFE strategy brings additional benefits to communities: improving quality of life by preventing further vision loss with surgery, improving access to water, sanitation and hygiene practices and providing an annual dose of azithromycin that has been shown to reduce child mortality from malaria, diarrhoea and bacterial respiratory tract infections. Through the SAFE strategy, the trachoma community will continue to work with health and development communities to achieve equitable access to eye health care and universal health coverage for all.
